# Early targeted next-generation sequencing accelerates clinical recovery in children with community-acquired pneumonia: A retrospective study

**DOI:** 10.12669/pjms.42.6.14330

**Published:** 2026-06

**Authors:** Di Lian, Dong Wang, Chenye Lin, QiuYu Tang

**Affiliations:** 1 Di Lian, MMed, College of Clinical Medicine for Obstetrics and Gynecology and Pediatrics, Fujian Medical University, China; 2 Dong Wang, MMed, Fujian Children’s Hospital, (Fujian Branch of Shanghai Children’s Medical Center), Fuzhou, Fujian 350014, China; 3 Chenye Lin, MMed, Fujian Children’s Hospital, (Fujian Branch of Shanghai Children’s Medical Center), Fuzhou, Fujian 350014, China; 4 QiuYu Tang, MMed, College of Clinical Medicine for Obstetrics and Gynecology and Pediatrics, Fujian Medical University, China

**Keywords:** Community-acquired pneumonia, Children, Clinical outcome, Diagnostic value, Economic value, Targeted next-generation sequencing (tNGS)

## Abstract

**Background & Objective:**

The clinical impact of testing timing for targeted next-generation sequencing (tNGS) in pediatric community-acquired pneumonia (CAP) is poorly understood. We hypothesized that early tNGS implementation accelerates clinical recovery and evaluated its comprehensive value.

**Methodology:**

In a retrospective study at Fujian Children’s Hospital (2021-2023), 199 hospitalized children with CAP (BALF tested) were analyzed. Patients were grouped by tNGS vs. conventional testing (CT), with the tNGS group stratified into early (≤48h) and late (>48h) testing. We compared diagnostic yield, clinical outcomes, therapeutic adjustments, and costs.

**Results:**

tNGS demonstrated significantly higher sensitivity than CT for both bacteria (86.0% vs. 19.0%, P<0.001) and viruses (88.1% vs. 26.2%, P<0.001), and excelled at identifying mixed infections (55.8%). Critically, the early-testing group showed significantly shorter times to cough improvement (median 6.0 vs. 9.0 days, P=0.002) and rale resolution (median 7.0 vs. 8.0 days, P=0.027) compared to the late-testing group. This was associated with more timely therapeutic adjustments (P<0.05). A non-significant trend toward lower costs and shorter hospital stays was also observed.

**Conclusion:**

Early tNGS implementation in pediatric CAP not only enhances diagnostic yield but, more importantly, accelerates clinical recovery by facilitating timely, targeted therapeutic interventions. These findings provide strong evidence for integrating early tNGS into clinical workflows, marking a shift from simple pathogen identification to an outcome-driven diagnostic strategy.

## INTRODUCTION

Community-acquired pneumonia (CAP) persists as a predominant cause of pediatric morbidity and mortality globally, exerting significant strain on healthcare infrastructures and familial resources.^1^,^2^ The precise and timely identification of etiological agents is imperative for the implementation of targeted antimicrobial interventions. Nevertheless, conventional diagnostic methodologies, including culture-based techniques and serological assays, are frequently constrained by suboptimal detection rates, protracted processing durations, and restricted pathogen spectrum.^3^,^4^ These limitations often necessitate empirical antibiotic administration, thereby exacerbating antimicrobial resistance and potentially delaying appropriate therapeutic interventions.

Targeted next-generation sequencing (tNGS) has recently emerged as a transformative diagnostic tool, addressing critical limitations of conventional methods through its enhanced sensitivity and comprehensive pathogen detection capabilities.^5^ Its utility in simultaneously identifying diverse bacterial and viral pathogens has been well-documented in respiratory infections.^6^,^7^ Although the diagnostic superiority of tNGS is increasingly established, a significant knowledge gap persists regarding its clinical implications, particularly the influence of testing timing on patient outcomes. Previous investigations, including our prior work, have primarily focused on evaluating the diagnostic yield of tNGS across various sample types or comparing its performance with alternative molecular assays. The current challenge lies not in demonstrating the pathogen detection capacity of tNGS, but in elucidating how and when its application can optimize clinical decision-making and improve patient outcomes.^8^

To address these critical questions, we conducted a retrospective study with two primary objectives:

- To validate the diagnostic utility of tNGS in pediatric community-acquired pneumonia (CAP).
- To investigate two novel clinical dimensions: the hypothesis that early tNGS implementation (within 48 hours of admission) accelerates clinical recovery, and the impact of early testing on subsequent therapeutic modifications.

Through this analysis, we aim to provide evidence-based insights for the strategic integration of tNGS into pediatric CAP management, advancing its application from theoretical validation to practical implementation in clinical workflows.

## METHODOLOGY

A retrospective cohort study was conducted at Fujian Children’s Hospital, a tertiary pediatric medical center located in Southeast China. The study population comprised hospitalized children diagnosed with CAP between December 2021 to August 2023 who had undergone BALF examination.

### Inclusion criteria:

- A confirmed diagnosis of CAP in accordance with the guidelines established by the Pediatric Branch of the Chinese Medical Association,
- An age range of 28 days to 18 years.

### Exclusion criteria:

- included patients with non-infectious pneumonia, hospital-acquired pneumonia, or incomplete medical records.

### Ethical Approval:

The study protocol was approved by the Institutional Review Board of Fujian Children’s Hospital (approval number: 2022ETKLR10019; dated October 1, 2022), and the requirement for individual informed consent was waived due to the retrospective, observational nature of the study and the use of anonymized data.

### Data collection:

We collected the following data from the electronic medical records system:

- *Demographics and Baseline Data:* Age, sex, disease severity (mild, severe, or critical), and underlying comorbidities.
- *Pathogen Detection Results:* Results from both tNGS and conventional tests (including sputum culture, blood culture, serological tests for Mycoplasma pneumoniae, and viral antigen/antibody panels).
- *Clinical Outcomes:* Time to improvement of major symptoms (cough, fever), time to disappearance of pulmonary rales, length of hospital stay (LOS), and incidence of complications.
- *Therapeutic Interventions:* Use of antibiotics and interferons, and any adjustments to antimicrobial therapy within 72 hours of admission.
- *Economic Data: Total* hospitalization costs and antimicrobial drug costs.
- tNGS and Conventional Testing

BALF samples were collected and processed in accordance with standardized protocols. For tNGS, DNA and RNA were extracted, followed by library preparation using a commercially available kit (GenCap, China) designed to identify 153 common respiratory pathogens. Sequencing was performed on the Illumina MiniSeq platform. Conventional diagnostic tests were conducted following established laboratory protocols.

### Statistical analysis:

Statistical analyses were conducted using R software (version 4.2.1). Continuous variables were summarized as median (interquartile range, IQR) and compared using the Wilcoxon rank-sum test. Categorical variables were expressed as frequencies (percentages) and analyzed using the chi-square test or Fisher’s exact test, as appropriate. A two-sided *P*-value < 0.05 was considered statistically significant.

## RESULTS

A total of 199 children diagnosed with Community-Acquired Pneumonia (CAP) were included in this retrospective study. The baseline characteristics of the cohort are summarized in Table-I. The median age of the patients was 1.5 years (Interquartile Range [IQR], 0.5-6.0 years), with a slight male predominance (58.3%). A significant portion of the cohort presented with severe or critical illness (51.7%), and 48 patients (24.1%) had underlying comorbidities.

**Table-I:** Baseline Characteristics of the 199 Children with Community-Acquired Pneumonia.

Characteristic	Category	N (%)
***Age Group (years)***		
	≤1	87 (43.72)
	>1 to 5	43 (21.61)
	>5	69 (34.67)
Gender		
	Male	116 (58.30)
	Female	83 (41.70)
***Disease Severity***		
	Mild	96 (48.24)
	Severe (non-critical)	51 (25.63)
	Critical	52 (26.13)
***Underlying Comorbidities***		
	Present	48 (24.12)
	Absent	151 (75.88)

Data are presented as n (%).

**Fig.1:**
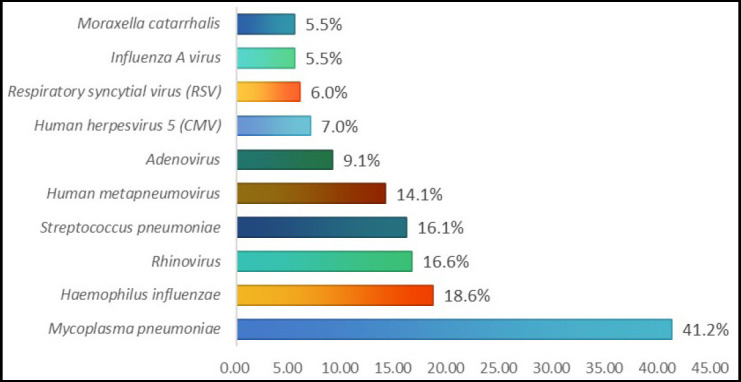
Distribution of the ten most common pathogens detected by tNGS in 199 children with community-acquired pneumonia (CAP). The bar chart illustrates the detection rates of the top ten pathogens identified from bronchoalveolar lavage fluid (BALF) samples. The y-axis represents the specific pathogen, and the x-axis represents the detection rate (%). Abbreviations: RSV, respiratory syncytial virus; CMV, cytomegalovirus.

Using tNGS, at least one potential pathogen was identified in 177 (88.9%) of the 199 patients. The ten most frequently detected pathogens are presented in Fig.1. *Mycoplasma pneumoniae* was the most common pathogen, identified in 41.2% of cases, followed by *Haemophilus influenzae* (18.6%) and Rhinovirus (16.6%). Notably, a significant proportion of identified pathogens were viruses, highlighting the importance of virological diagnosis in pediatric CAP.

**Fig.2:**
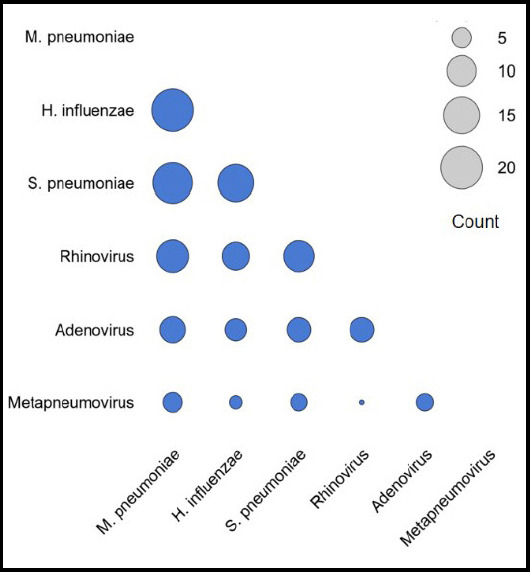
Co-infection patterns of the six most common pathogens detected by tNGS. The bubble plot illustrates the frequency of co-infections between pairs of the top six pathogens identified in the study cohort. The y-axis represents the first pathogen in a pair, and the x-axis represents the second. The size of each bubble is directly proportional to the number of patients in whom that specific pair of pathogens was co-detected. The legend on the right provides a scale for interpreting the bubble size in terms of the number of co-infection cases.

Mixed infections were highly prevalent, identified in 111 (55.8%) patients. To visualize the most significant co-infection patterns, a bubble plot was constructed for the six most common pathogens (Fig.2). The highest frequency of co-infection was observed between *M. pneumoniae* and *H. influenzae* (n=20), followed by *M. pneumoniae* and *S. pneumoniae* (n=18).

The diagnostic performance of tNGS was significantly superior to that of conventional tests (CTs) for the detection of bacteria and viruses. As detailed in Table-II, tNGS demonstrated a markedly higher sensitivity for bacterial detection compared to sputum culture (86.0% vs. 19.0%, P < 0.001). Similarly, for viral detection, the sensitivity of tNGS (88.1%) was substantially greater than that of the seven virus antigen panel (26.2%, P < 0.001). The diagnostic performance of tNGS for atypical pathogens varied depending on the pathogen type. The sensitivity of tNGS for *M. pneumoniae* was significantly higher than the colloidal gold method but lower than the indirect hemagglutination assay (IHA). In the detection of fungi, no significant difference in performance was observed between tNGS and the (1,3)-β-D-glucan test.

**Table-II:** Diagnostic Performance of tNGS versus Conventional Tests for Major Pathogen categories.

Pathogen Category	Test Method	Positive / Total	Sensitivity (%)	P-value
Bacteria	tNGS	86 / 100	86	<0.001
	Sputum Culture	19 / 100	19	
Viruses	tNGS	37 / 42	88.1	<0.001
	7-Virus Antigen Panel	11 / 42	26.2	
M. pneumoniae	tNGS	55 / 82	67.1	<0.001*
	Colloidal Gold	17 / 84	20.2	
	IHA (titer ≥1:160)	54 / 110	49.1	
Fungi	tNGS	9/30	30	1.000
	(1,3)-β-D-Glucan Test	8/30	26.7	

* P-value reflects the comparison between tNGS and the most commonly used conventional test in this cohort (IHA). Abbreviations: tNGS, targeted next-generation sequencing; IHA, indirect hemagglutination assay.

To evaluate the impact of testing timeliness, patients who underwent tNGS were stratified into an early-testing group (≤ 48 hours of admission, n=55) and a late-testing group (> 48 hours, n=67). As shown in Table-III, patients in the early-testing group experienced significantly faster clinical improvement. The median time to cough resolution was shorter in the early group compared to the late group (6.0 days vs. 9.0 days, P = 0.002), and a similar significant difference was observed for the time to disappearance of pulmonary rales (7.0 days vs. 8.0 days, P = 0.027).

**Table-III:** Clinical and Economic Outcomes in Patients Stratified by Timing of tNGS Testing.

Outcome	Early tNGS Group (n=55)	Late tNGS Group (n=67)	P-value
Clinical Improvement			
Time to Cough Resolution (days), median [IQR]	6.0 [5.5-10.5]	9.0 [7.0-12.0]	0.002
Time to Disappearance of Pulmonary Rales (days), median [IQR]	7.0 [5.5-8.0]	8.0 [6.5-10.5]	0.027
Therapeutic Intervention (within 72h of admission)			
Appropriate Antibiotic Adjustment, n (%)	30 (54.6)	21 (31.3)	0.016
Interferon Therapy, n (%)	28 (50.9)	19 (28.4)	0.018
Economic Indicators			
LOS (days), median [IQR]	8.0 [6.0-9.0]	9.0 [7.0-11.0]	0.203
Total Hospitalization Costs (CNY), median [IQR]	8,392 [6,852-12,186]	9,697 [7,408-13,235]	0.218

Data are presented as median [interquartile range (IQR)] or n (%). Abbreviations: tNGS, targeted next-generation sequencing; IQR, interquartile range; CNY, Chinese Yuan.

This clinical benefit was associated with more rapid therapeutic interventions. A significantly higher proportion of patients in the early-testing group received appropriate antibiotic adjustments (54.6% vs. 31.3%, P = 0.016) and interferon therapy (50.9% vs. 28.4%, P = 0.018) within the first 72 hours of admission. Regarding economic outcomes, while not statistically significant, a favorable trend was observed in the early-testing group, with a shorter median length of hospital stay (8.0 vs. 9.0 days) and lower median total hospitalization costs (8,392 vs. 9,696 CNY).

## DISCUSSION

This study systematically evaluated the diagnostic, clinical, and economic implications of tNGS in pediatric CAP, with particular emphasis on the temporal aspects of its clinical deployment. Our empirical data not only demonstrate the diagnostic superiority of tNGS over conventional microbiological methods but, more significantly, substantiate our central hypothesis that early implementation of tNGS facilitates expedited clinical recovery and enables more precise therapeutic modifications. These findings extend beyond technological validation, offering a comprehensive framework for optimizing the clinical integration of tNGS in pediatric respiratory infections.

Our results clearly demonstrate the significant advantages of tNGS in pathogen detection. tNGS identified at least one pathogen in 88.9% of cases, far exceeding the diagnostic yield of conventional tests such as sputum culture (19%) and viral antigen panels (26.2%).^9^-^11^ The sensitivity of tNGS for bacterial detection was 86%, compared to just 19% for sputum culture, which is consistent with findings from previous studies in both pediatric and adult populations.^12^ For viral pathogens, tNGS showed a sensitivity of 88.1%, markedly higher than the 26.2% sensitivity of the seven virus antigen panel. This performance is consistent with a growing body of literature that has demonstrated the superior diagnostic capability of tNGS,^13^ particularly in identifying a broad range of pathogens, including bacteria, viruses, and atypical pathogens like *Mycoplasma pneumoniae* and fungi.^14^,^15^ In our cohort, the most common pathogens detected by tNGS included *Mycoplasma pneumoniae*, *Haemophilus influenzae*, and rhinoviruses, with *Mycoplasma pneumoniae* accounting for 41.2% of infections. This is consistent with prior studies that have identified *M. pneumoniae* as a leading pathogen in pediatric CAP.^16^,^17^ Additionally, mixed infections were detected in 55.8% of cases, underscoring the complexity of CAP in children and the limitations of conventional diagnostic methods.^18^ The high frequency of mixed infections highlights the critical role of advanced diagnostic technologies like tNGS, which can simultaneously detect multiple pathogens, thereby providing a more comprehensive and accurate diagnosis.^19^

One of the key findings of this study is the impact of early tNGS testing on clinical outcomes. Patients who underwent early tNGS testing (within 48 hours of admission) experienced significantly faster clinical improvement, including a shorter time to cough resolution and faster disappearance of pulmonary rales, compared to those who underwent testing later. These findings align with previous studies that have shown the benefits of early pathogen identification in improving clinical outcomes in CAP.^20^ The faster resolution of symptoms in the early-testing group was likely due to more timely and appropriate therapeutic interventions. Early identification of pathogens enabled clinicians to adjust antibiotic therapy more rapidly, with 54.6% of early-testing patients receiving appropriate antibiotic adjustments within 72 hours of admission, compared to only 31.3% in the late-testing group. This finding underscores the importance of rapid and accurate diagnostic testing in guiding antimicrobial therapy, which is particularly critical in pediatric patients where the risk of adverse outcomes from delayed or inappropriate treatment is high.^21^ While our study cohort consisted of hospitalized patients, the implications for outpatients with CAP are significant. Early tNGS in high-risk outpatients could potentially prevent unnecessary hospitalizations by enabling immediate targeted therapy, thereby reducing the socioeconomic burden on families.

While the clinical benefits of early tNGS testing were evident, the economic implications were more complex. Although the early-testing group showed a favorable trend toward shorter hospital stays and lower total hospitalization costs, these differences did not reach statistical significance. The median length of stay was 8.0 days in the early-testing group versus 9.0 days in the late-testing group, and the median total hospitalization costs were lower in the early-testing group (8,392 CNY vs. 9,696 CNY). These trends are consistent with findings from other studies that have suggested a potential economic benefit of early tNGS testing, but the high upfront costs of tNGS may offset these savings.^22^

The lack of statistical significance in the economic outcomes may be attributed to the relatively small sample size and the retrospective nature of the study. In addition, the cost-effectiveness of tNGS will depend on several factors, including the cost of the test, the clinical setting, and the ability to reduce the duration of hospitalization and antimicrobial therapy. A recent systematic review concluded that while NGS shows great clinical promise, robust evidence for its cost-effectiveness in pneumonia remains limited.^23^

### Strengths of the study:

This study uniquely quantifies the clinical impact of tNGS timing in pediatric CAP. Unlike prior research focused solely on diagnostic yield, we demonstrated that early implementation (≤48 hours) directly correlates with faster resolution of cough and pulmonary rales. By defining this ‘golden window,’ our findings provide actionable evidence for clinicians to optimize diagnostic workflows.

### Limitations:

First, its retrospective, single-center design limits generalizability, as findings from Fujian Children’s Hospital may not apply to diverse settings. Second, non-standardized tNGS ordering may introduce selection bias, favoring severe cases. Third, while the sample size detected clinical differences, it was insufficient for economic outcomes like costs. Finally, the lack of long-term follow-up restricts assessment of sustained impacts.

## CONCLUSION

Our investigation demonstrates that early implementation of tNGS significantly enhances diagnostic accuracy and, more importantly, facilitates expedited clinical recovery through the prompt initiation of targeted therapeutic strategies. Although comprehensive cost-effectiveness analyses necessitate further validation, our results strongly support the strategic incorporation of early tNGS into the clinical management of pediatric CAP, particularly in severe or complex presentations. This paradigm represents a critical transition from diagnostic identification to active enhancement of clinical outcomes, thereby advancing the application of tNGS from theoretical validation to practical, outcome-driven integration within clinical practice.

### Recommendations:

Future studies should address the current limitations through multicenter, prospective randomized controlled trials (RCTs) with standardized protocols and larger samples. These trials are essential to validate our findings and must incorporate robust cost-effectiveness analyses to justify the high upfront costs of tNGS against the potential clinical and economic savings derived from reduced hospital stays and optimized antibiotic use.

### Authors’ Contribution:

**DL, DW, QT:** Literature search, Conceptualization and Study Design.

**DW, CL DL:** Data Acquisition. Analysis and Interpretation of Data.

**DL, DW:** Drafting the Manuscript. Equal Contribution. contributed equally to this work and should be considered co-first authors.

**QT:** Literature search, Critical Revision for analysis.

All authors have read the final version of the manuscript and are also responsible for the integrity of the study.
